# Hif-2α regulates lipid metabolism in alcoholic fatty liver disease through mitophagy

**DOI:** 10.1186/s13578-022-00889-1

**Published:** 2022-12-07

**Authors:** Mei-fei Wu, Guo-dong Zhang, Tong-tong Liu, Jun-hao Shen, Jie-ling Cheng, Jie Shen, Tian-yu Yang, Cheng Huang, Lei Zhang

**Affiliations:** 1 School of Pharmacy, Inflammation and Immune Mediated Diseases Laboratory of Anhui Province, Anhui Medical University, Hefei, 230032 China; 2 Sixth People’s Hospital South Campus, Shanghai Jiaotong University, Shanghai, 201400 China; 3 The Key Laboratory of Anti-Inflammatory and Immune Medicines, Ministry of Education, Anhui Medical University, Hefei, 230032 China

**Keywords:** Hif-2α, Fatty acid β-oxidation, BNIP3, Mitophagy, AFLD

## Abstract

**Background:**

Disordered lipid metabolism plays an essential role in both the initiation and progression of alcoholic fatty liver disease (AFLD), and fatty acid β-oxidation is increasingly considered as a crucial factor for controlling lipid metabolism. Hif-2α is a member of the Hif family of nuclear receptors, which take part in regulating hepatic fatty acid β-oxidation. However, its functional role in AFLD and the underlying mechanisms remain unclear.

**Results:**

Hif-2α was upregulated in EtOH-fed mice and EtOH-treated AML-12 cells. Inhibition or silencing of Hif-2α led to increased fatty acid β-oxidation and BNIP3-dependent mitophagy. Downregulation of Hif-2α activates the PPAR-α/PGC-1α signaling pathway, which is involved in hepatic fatty acid β-oxidation, by mediating BNIP3-dependent mitophagy, ultimately delaying the progression of AFLD.

**Conclusions:**

Hif-2α induces liver steatosis, which promotes the progression of AFLD. Here, we have described a novel Hif-2α-BNIP3-dependent mitophagy regulatory pathway interconnected with EtOH-induced lipid accumulation, which could be a potential therapeutic target for the prevention and treatment of AFLD.

**Supplementary Information:**

The online version contains supplementary material available at 10.1186/s13578-022-00889-1.

## Introduction

Alcohol consumption is a social practice in many countries. As the liver is the key site for alcohol metabolism, the morbidity and mortality of alcoholic liver disease (ALD) induced by alcohol consumption are serious problems [1, 2]. Alcoholic fatty liver disease (AFLD) is the initial stage of ALD and is characterized by mild liver inflammation and damage caused by hepatic lipid accumulation. AFLD pathogenesis occurs via ethanol-induced changes in enzyme activity, including mitochondrial dysfunction, oxidative stress and inflammatory cytokines, which lead to abnormal lipid metabolism in hepatocytes [3, 4]. Persistent hepatic steatosis can progress to more serious disease stages, such as alcoholic steatohepatitis (ASH), alcoholic fibrosis and hepatocellular carcinoma (HCC) [5]. Although steatosis is involved in the pathogenesis of AFLD, its regulatory mechanism remains unclear.

Hepatic lipid deposition is attributed to an imbalance between lipid acquisition and lipid clearance [6]. Fatty acid β-oxidation is the main determining factor in lipid clearance, abnormal lipid accumulation and lipotoxicity in acute and chronic kidney diseases results from a pattern of severe deficiency of fatty acid β-oxidation [7, 8]. Fatty acid β-oxidation is mediated by several signaling pathways, including PPAR-α/PGC-1α [9, 10]. The interrelationship between PGC-1α and PPAR-α controls the expression of key enzymes involved in fatty acid uptake and β-oxidation pathways [11, 12]. In PPAR-α deficient mice, chronic ethanol feeding is associated with progressive intrahepatic triglyceride accumulation, due to reduced β-oxidation and alterations to the tricarboxylic acid cycle [6, 13]. The liver is also protected by mitophagy, which is a specific kind of organellophagy that is responsible for the clearance of damaged mitochondria. A recent study showed that the loss of mitophagy results in the development of liver-associated diseases, including liver injury, fatty liver disease (FLD), viral hepatitis and liver cancer [14–16]. However, it has not yet been clarified whether mitophagy can improve fatty acid β-oxidation through the PPAR-α/PGC-1α signaling pathway during the progression of AFLD.

Hypoxia inducible factors (Hifs) are core regulators that are widespread in mammals under conditions of hypoxia [17]. Accumulating evidence suggests that acute and chronic alcohol consumption increases hepatic oxygen consumption, resulting in pericentral hypoxia in the liver and the activation of Hif pathways [18, 19]. Hif-2α activity is thought to be the primary factor driving the adjustment of hepatic lipid metabolism, as Hif-2α is involved in the regulation of many key enzymes in fatty acid β-oxidation, including carnitine-palmitoyl transferase-1 (CPT-1) [20] Given the pivotal role of Hif-2α in lipid metabolism, and the intimate relationship between FLD and mitophagy, in the present study we aimed to investigate whether Hif-2α can induce steatosis in AFLD by interfering with mitophagy activity.

In the present study, we first measured the levels of Hif-2α, both in vitro and in vivo, to establish its involvement during alcohol-induced liver disease. We generated knockdown Hif-2α in hepatocyte cells to study the role of Hif-2α following ethanol consumption, and performed mechanistic studies to determine its role in the PPAR-α/PGC-1α signaling pathway and hepatic fatty acid β-oxidation in order to clarify the role of mitophagy during AFLD. These data may potentially provide new therapeutic targets for liver steatosis.

## Materials and methods

### Animal studies

Male C57B6/L mice (8 weeks old) were purchased from the Experimental Animal Center of Anhui Medical University (Hefei, China). Mice were randomly divided into four groups, the control group (CD-fed), model group (EtOH-fed), blank solvent group, and PT2399 treatment group (n = 10 per group). All experiments were performed according to the institutional ethical guidelines for laboratory animal care and use of Anhui Medical University. An AFLD model was established using the National Institute on Alcohol Abuse and Alcoholism (NIAAA)- recommended method of a Lieber-De Carli (LD) liquid diet and alcohol intragastric administration. Animal feed was obtained from TROPHIC Animal Feed High-Tech Co., Ltd. (Hai’ an, Jiangsu, China). The modeling process lasted a total of 16 days, including liquid diet adaptation (5 days), modeling (10 days), intragastric administration (1 time), and specimen acquisition stages (1 day). Mice in the EtOH-fed group were randomly fed a LD liquid diet containing 5% ethanol (vol/vol) for 10 days. Mice in the PT2399 treatment group received PT2399 (10 mg/kg) twice daily from day 10‒17 via intragastric administration. Mice were anesthetized 9 h after the final oral gavage of 33% ethanol (5 g/kg), and the blood and liver tissue samples were collected for subsequent analysis. At least six independent experiments were performed.

### Morphological assessment

Liver tissue samples were fixed with 4% paraformaldehyde for 24 h, and then embedded in paraffin blocks and stained with hematoxylin and eosin (H&E). Immunohistochemistry (IHC) was performed according to a standard procedure. Brown staining was considered as a positive result. Images were captured using a Panoramic 250 slide scanner (3DHistech Ltd).

### Biochemical assays

The activities of serum alanine aminotransferase (ALT) and aspartate aminotransferase (AST), and the levels of triglycerides (TGs) and total cholesterol (T-CHO) were measured using commercial assay kits (Jiancheng, Nanjing, China) in a microplate reader (Biotek, USA) at the appropriate wavelength.

### Transmission electron microscopy (TEM)

Grain size liver tissue samples were fixed in 2.5% glutaraldehyde at 4 °C and then in 1% osmium tetroxide for 4 h on ice. Ultrathin Sects. (60 nm thick) were cut on an Ultracut E Microtome (Reichert‐Jung; Leica Microsystems, Wetzlar Germany) with a diamond knife (Diatome, Hatfield, PA, USA), collected on copper grids, and stained with uranyl acetate and lead citrate. Sections were dehydrated sequentially in a graded series of ethanol, infiltrated with graded mixtures of propylene oxides, embedded in fresh resin, and incubated at 60 °C for 48 h. Electron micrographs were obtained using TEM (Hitachi, Japan).

### Cell culture

Alpha mouse liver 12 (AML-12) cells were purchased from the Type Culture Collection of the Chinese Academy of Sciences (Shanghai, China). Cells were maintained in Dulbecco’s modified Eagle’s medium (DMEM, Gibco, Carlsbad, USA) supplemented with 10% fetal bovine serum (FBS, Hangzhou Sijiqing Biological Engineering Materials, China) and 1% penicillin–streptomycin (Biyuntian, China) at 37 °C under an atmosphere of 5% CO_2_. The medium was changed once a day. They were treated with 100 mM ethanol for 48 h. At least three independent replicates were performed for each experiment.

### Oil red o staining

Cultured cells were fixed in 4% paraformaldehyde for 15 min. Sections were then stained with Oil red o for 30 min, and then washed with 60% dimethylcarbinol and double distilled water. The fat drops were observed using an inverted fluorescent microscope. The same procedure was performed for fixed liver tissue samples.

### Cell transfection

Hif-2α siRNA plasmid and BNIP3 shRNA plasmid (Gene Pharma, Shanghai, China) were designed to downregulate the expression of Hif-2α and BNIP3, respectively. pc-DNA3.1-BNIP3 plasmid was designed to upregulate the expression of BNIP3. Transfection was performed using LipofectamineTM2000 (Invitrogen, Carlsbad, MA), according to the manufacturer’s instructions. After 6 h of transfection, the medium was changed to DMEM and ethanol was added. The following primers were used: siRNA-Hif-2α (mouse): 5′-GCAACUACCUGUUCACCAATT-3′ (sense) and 5′-UUGG UGAA CAG GUAGUUG CT-3′ (antisense).

### Cell viability assay

Cultured cells were seeded in a 96-well plate, and 20 µL of 3-(4,5-dimethylthiaz ol-2-yl)-2,5-diphenyltetrazoliumbromide (MTT, Sigma–Aldrich) was added to each well and then incubated for 4 h. Thereafter, 100 µL of dimethylsulfoxide (DMSO, Sigma-Aldrich) was added to each well and incubated for 10 min. Cell viability was determined by measuring the absorbance (A) at 490 nm using a spectrophotometer (BioTek Instruments, USA).

### RNA extraction and quantitative real-time PCR (qRT–PCR)

Total cellular RNA was isolated using TRIzol Reagent (Invitrogen, USA) according to the manufacturer’s instructions. Reverse transcription of total RNA was performed using a kit. PCR was performed using the PIKO REAL 96 (Thermo Fisher Scientific) system. Real-time quantitative PCR (qRT-PCR) analysis was performed using SYBR® Prime Script™ RT–PCR Kit (TAKARA, Kusatsu, Japan). β-actin was used as the reference gene. The relative expression of each gene was calculated using the 2^−ΔΔCt^ method. The specific sequences of each primer were as follows:

**Table:** 

Names	Upstream primer sequence (5ʹ–3ʹ)	Downstream primer sequence (5ʹ–3ʹ)
β-actin	CACATGCGATACGTCTTT	CTCGCACCCACGCTCACACA
Beclin1	GATGGAAGGGTCTAAGACGTCCAA	TTTCGCCTGGGCTGTGGTAAG
BNIP3	GCATGAGTCTGGACGGAGTAG	CCGACTTGACCAATCCCATA
CPT-1α	CCTCCGTAGCTGACTCGGTA	GGAGTGACCGTGAACTGAAA
Hif-2α	CTCAGTGGGAGCGACTCTTCA	GGCCTCTGTGGTACACGACAA
LC3B	CTAACTGCCACTTCAACC	CAGACTTCCTGCTACGC
MCAD	TAATCGGTGAAGGAGCAGGTTT	GGCATACTTCGTGGCTTCGT
PGC-1α	AACAATGAGCCTGCGAAC	CCTCGTTGTCAGTGGTCA
PPAR-α	GGCCAACTATGGTGGACATCA	ACCAATCTGGCTGCTGCACGAA

### Western blotting

Liver tissue samples and cultured cells were lysed in RIPA buffer. The nuclear and cytoplasmic protein fractions were extracted using a nuclear and cytoplasmic protein extraction kit (Beyotime, Shanghai, China) according to the manufacturer’s protocol. Protein concentration was measured using a BCA protein assay kit (Boster, Wuhan, China). The protein samples were separated by SDS–PAGE (Bio–Rad Laboratories Inc., Berkeley, CA, USA) and transferred onto polyvinylidene fluoride (PVDF) membranes (Millipore Corp, Billerica, MA, USA). The PVDF membranes were incubated with primary antibodies against PGC-1α, BNIP3, Beclin-1, MCAD, CPT-1α (1:500; Proteintech, Wuhan, China), PPAR-α (1:500; Cell Signaling Technology, USA), Hif-2α and LC3B (1:1000; Abcam, Cambridge, MA, USA), and Lamin A/C (1:500; Zhongshan Jinqiao, China) for 24 h, and then incubated with secondary antibody (1:500; Zhongshan Jinqiao, China) for 1 h at room temperature. The protein bands were visualized by enhanced chemiluminescence (ECL) assay (Thermo Scientific, USA), and the blots were analyzed using Image Lab 3.0 (Bio–Rad, Hercules, CA, USA). The gray value of protein bands was calculated using Image J 1.50i (National Institutes of Health, USA) and normalized to that of the internal control, β-actin.

### Laser scanning confocal microscopy

The fluorescence of intracellular Fluo-3 was measured by confocal laser scanning fluorescence microscopy (Carl-Zeiss, Jena, Germany) at an excitation wavelength of 488 nm and an emission wavelength of 525 nm. Grayscale images were collected at different time points from 0 to 5 min, and then archived as TIFF image files. Images were analyzed using Leica-sp5 software from the Leica Application Suite (LAS) AF software (Leica Microsystems Inc., Buffalo Grove, IL, USA).

### Immunofluorescence

Cultured cells were fixed in 4% formaldehyde for 15 min, and then blocked with 10% bovine serum albumin (BSA, Sigma–Aldrich) to prevent nonspecific staining. Cells were incubated with primary antibodies against Hif-2α or BNIP3 (1:100) at 4 ℃ overnight, and then probed with Fluorescein isothiocyanate (FITC)-conjugated anti-rabbit or anti-mouse IgG (Molecular Probes, Beijing, China) at 37 °C for 1 h. Nuclear staining was achieved by incubating cells with 4′,6-diamidino-2-phenylindole and dilactate (DAPI; Invitrogen, Carlsbad, CA, USA). Stained sections were examined using a Carl-Zeiss microscope.

### Monitoring of mitophagy

To detect mitophagy, cultured cells were stained with 150 nM Mito Tracker Red (MTR, Invitrogen, L7528) in combination with LC3B. Stained cells were visualized using a Carl-Zeiss microscope within 24 h after mounting.

### Statistical analysis

Data are presented as the mean ± standard deviation (SD). A Student’s t-test or one way ANOVA was used to assess statistically significant differences between groups in GraphPad Prism software. Significance was set at p < 0.05. Results are representative of at least three independent experiments.

## Results

### Upregulation of Hif-2α in vivo and in vitro

**Fig. 1:**
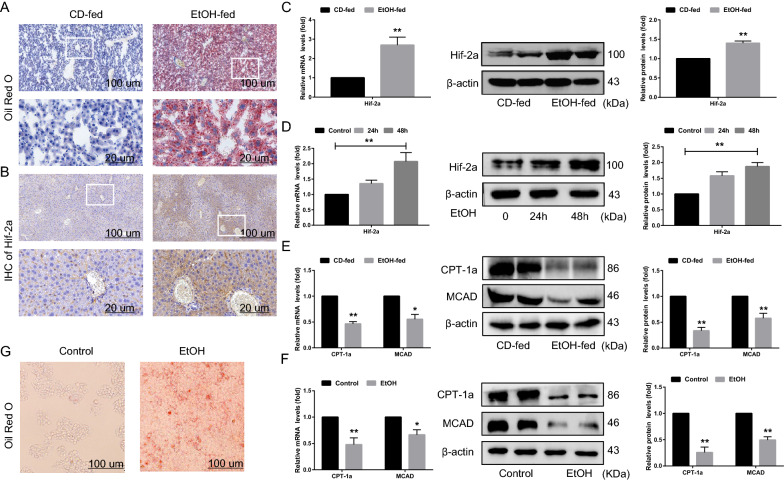
Upregulation of Hif-2α in vivo and in vitro. C57 male mice were fed an EtOH liquid diet or the control diet for 16 days. AML-12 cells were treated with 100 mM EtOH for 48 h. **A**, **B** Representative Oil red o staining images and IHC-Hif-2α of liver tissue samples from EtOH-fed mice (scale bar = 100 μm or 20 μm) (n = 6). **C**, **D** qRT-PCR and western blotting analysis of Hif-2α and **E**, **F** CPT-1α and MCAD expression in EtOH-fed mice (n = 6) and EtOH-treated AML-12 cells (n = 3). **G** Representative Oil red o staining images of EtOH-treated AML-12 cells (scale bar = 100 μm) (n = 3). Data are presented as the mean ± S.D. *P < 0.05, **P < 0.01

To verify the involvement of Hif-2α in the pathogenesis of AFLD, we generated a mouse model of AFLD using the chronic-plus-binge feeding method in male C57B6/L mice [21]. As expected, compared to CD-fed mice, ethanol feeding resulted in exacerbated liver lipid droplet accumulation (Fig. 1A), increased liver-to-body ratio, and higher serum levels of AST, ALT, T-CHO, and TG (Additional file 1: Figure S1A), as well as increasing the degree of liver injury based on H&E staining (Additional file 1: Figure S1B).

Abnormally insufficient lipid metabolism is the main pathological feature of AFLD, and Hif-2α is critically relevant to lipid metabolism [6, 20]. In the present study, IHC (Fig. 1B), qRT–PCR, and western blotting consistently showed that Hif-2α was highly expressed in liver tissue from EtOH-fed mouse, and in AML-12 cells after treatment with 100 mM ethanol for 48 h (Fig. 1C, D), which was accompanied by lipid deposition (Fig. 1G, Additional file 1: Figure S2A). Immunofluorescence revealed that the intracellular distribution of Hif-2α in hepatocytes was elevated in the nucleus but decreased in the cytoplasm after ethanol stimulation, which was verified using western blotting (Additional file 1: Figure S1D). The expression of two key enzymes involved in hepatic fatty acid β-oxidation, CPT-1α and MCAD, was markedly downregulated in EtOH-fed mice and EtOH-treated AML-12 cells (Fig. 1E, F). Ethanol treatment therefore induces the upregulation of Hif-2α, both in vivo and in vitro. This prompted us to investigate the functional roles of Hif-2α during the occurrence of AFLD.

### Hif-2α inhibits fatty acid β-oxidation in EtOH-treated AML-12 cells and EtOH-fed mice

**Fig. 2:**
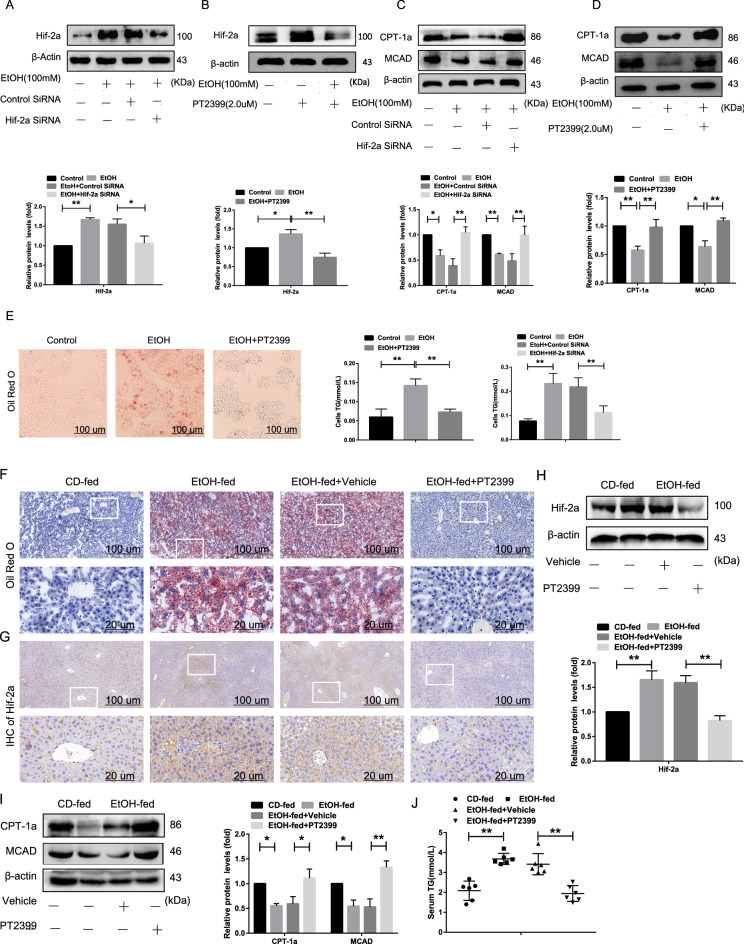
Hif-2α inhibits fatty acid β-oxidation in EtOH-treated AML-12 cells and EtOH-fed mice. EtOH-treated AML-12 cells were transfected with Hif-2α-siRNA or treated with PT2399 (2.0 μM) for 48 h, and mice fed EtOH liquid were intragastrically administered PT2399 (10 mg/kg) for 7 days. **A**, **B** Western blotting analysis of Hif-2α, and **C**, **D** CPT-1α and MCAD in Hif-2α-siRNA-treated and PT2399-treated AML-12 cells. **E** Representative Oil red o staining images of PT2399-treated AML-12 cells (scale bar = 100 μm), showing the triglyceride content in Hif-2α-siRNA- and PT2399-treated AML-12 cells (n = 3). **F**, **G** Representative Oil red o staining images and IHC-Hif-2α in PT2399-treated liver tissue (scale bar = 100 μm or 20 μm). **H**, **I** Western blotting analysis of Hif-2α, CPT-1α and MCAD in PT2399-treated liver tissue. **J** Serum triglyceride levels in PT2399-treated liver tissue (n = 6). Data are presented as the mean ± S.D. *P < 0.05, **P < 0.01

To investigate the potential connection between Hif-2α and fatty acid β-oxidation, EtOH-treated AML-12 cells were treated with Hif-2α-siRNA (Fig. 2A), and the Hif-2α antagonist PT2399 (2.0 µM) [22, 23] (Fig. 2B, Additional file 1: Figure S2B). As illustrated in Fig. 2C, D, there was a significant increase in MCAD and CPT-1α levels in the Hif-2α-siRNA and PT2399 treatment groups compared to that in the EtOH only group. Moreover, Hif-2α-siRNA and PT2399 treatment decreased the level of TG and the number of intracellular lipid droplets in EtOH-treated AML-12 cells (Fig. 2E). In comparison to that in the EtOH-fed mice, IHC (Fig. 2G) and western blotting consistently showed that the expression of Hif-2α decreased while the that of MCAD and CPT-1α increased after intragastric administration of PT2399 (10 mg/kg) for 7 days (Fig. 2H, I). H&E and Oil red o staining confirmed the loss of Hif-2α expression in hepatocytes treated with PT2399, verifying that the Hif-2α inhibitor efficiently decreased the number of liver lipid droplets and liver injury (Additional file 1: Figure S1C, Fig. 2F). The liver-to-body weight ratio, and the serum levels of ALT, AST, T-CHO, and TG in EtOH-fed mice decreased significantly after treatment with PT2399 (Additional file 1: Figure S2C, Fig. 2J). Depletion of Hif-2α expression may therefore increase hepatic fatty acid β-oxidation and subsequently alleviate lipid deposition, both in vivo and in vitro.

### Hif-2α inhibits BNIP3-dependent mitophagy in EtOH-fed mice and EtOH-treated AML-12 cells

**Fig. 3:**
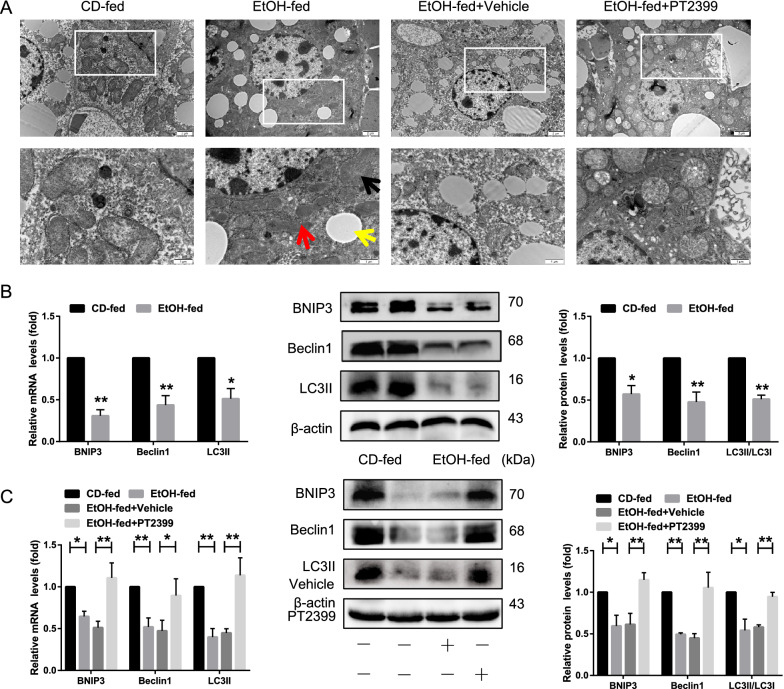

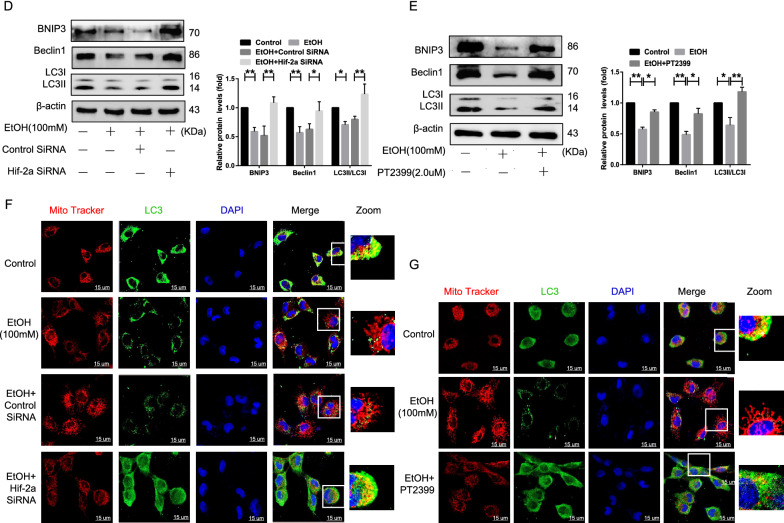
Hif-2α inhibits BNIP3-dependent mitophagy in EtOH-fed mice and EtOH-treated AML-12 cells. **A** Representative TEM images of lipid droplets (LDs), mitochondria, and autophagosomes in PT2399-treated liver tissue. Black arrows denote autophagosomes, yellow arrows denote LDs, and red arrows denote mitochondria. **B**, **C** qRT-PCR and western blotting analysis of BNIP3, Beclin1, and LC3II expression in liver tissue from EtOH-fed and PT2399-treated mice (n = 6). **D**, **E** Western blotting analysis of BNIP3, Beclin1, and LC3II/LC3I expression in Hif-2α-siRNA-treated and PT2399-treated AML-12 cells. Data are presented as the mean ± S.D. *P < 0.05, **P < 0.01. **F**, **G** Representative images of mitochondria (red), LC3 (green), and nuclei (blue) in Hif-2α-siRNA-treated and PT2399-treated AML-12 cells (scale bar = 15 μm) (n = 3)

Mitophagy, a type of selective autophagy, is a specific process that maintains lipid homeostasis in hepatocytes [14–16]. We next explored the effect of Hif-2α on mitochondrial function by evaluating mitochondrial morphology using electron microscopy in the livers of EtOH-fed mice. As shown in Additional file 1: Figure S3D, the mitochondria of CD-fed mice had a normal shape and active fission, whereas the mitochondria of EtOH-fed mice were enlarged with no observed fission. Fewer lipid vacuoles and autophagosomes were observed in EtOH-fed mice compared to those in CD-fed mice. PT2399 treatment also increased the number of lipid vacuoles and autophagosomes in EtOH-fed mice (Fig. 3A). These results suggest that Hif-2α increases liver steatosis and mitochondrial impairment following EtOH feeding, which are required for the progression of AFLD.

We also found that EtOH feeding inhibited the expression of mitophagy-related markers, including BNIP3, Beclin1 and LC3II in vivo, and this phenomenon was significantly reversed by the administration of PT2399 (Fig. 3B–C). We observed a concomitant decrease in the expression of mitophagy-associated proteins upon exposure to ethanol in vitro (Additional file 1: Figure S2D). Consistent with these findings, the colocalization of LC3 and Mito Tracker Red (MTR), in addition to the number of fluorescent puncta corresponding to BNIP3 and LC3 was decreased in EtOH-treated AML-12 cells (compared to the controls) (Additional file 1: Figure S3A, B). Silencing of Hif-2α upregulated the levels of BNIP3, Beclin1, and LC3II/LC3I, which was in agreement with the results obtained in the PT2399 treatment group (Fig. 3D–E). The colocalization of LC3 and MTR and the number of fluorescent puncta of BNIP3 and LC3 increased in response to Hif-2α-siRNA or PT2399 treatment (Fig. 3F–G, Additional file 1: Figure S3C). In contrast, co-transfection of Hif-2α-siRNA and BNIP3-shRNA into EtOH-treated AML-12 cells resulted in reduced BNIP3, Beclin1, and LC3II/LC3I expression (Additional file 1: Figure S2E). In summary, there may be a negative relationship between Hif-2α levels and mitophagy, and the loss of Hif-2α is sufficient to initiate mitophagy, both in vivo and in vitro, a phenomenon that could reduce lipid accumulation in the liver.

### Hif-2α regulates fatty acid β-oxidation through the PPAR-α/PGC-1α signaling via BNIP3-dependent mitophagy

**Fig. 4:**
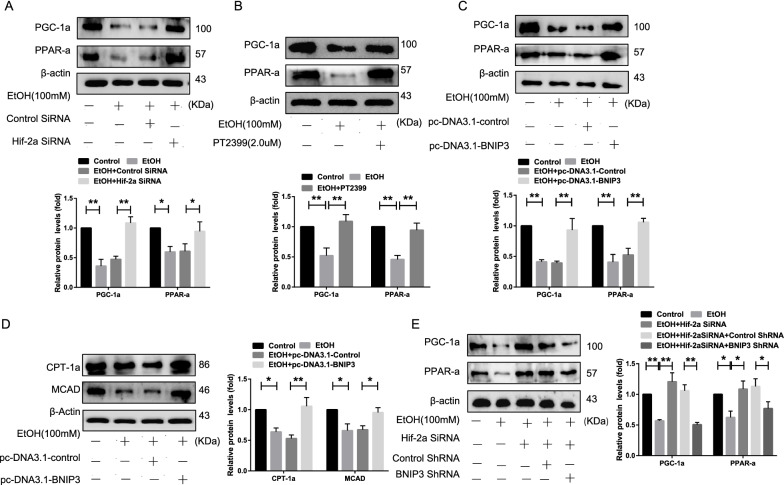
Hif-2α regulates fatty acid β-oxidation through the PPAR-α/PGC-1α signaling pathway via BNIP3-dependent mitophagy. **A**, **B**, **C** Western blotting analysis of PGC-1α and PPAR-α expression in Hif-2α-siRNA-treated, PT2399-treated, and pc-DNA3.1-BNIP3-treated AML-12 cells. **D** Western blotting analysis of CPT-1α and MCAD expression in pc-DNA3.1-BNIP3-treated AML-12 cells. **E** Western blotting analysis of PGC-1α and PPAR-α expression in Hif-2α-siRNA and BNIP3-shRNA co-treated AML-12 cells (n = 3). Data are presented as the mean ± S.D. *P < 0.05, **P < 0.01

Hepatic fatty acid β-oxidation is regulated by multiple signaling pathways, such as PPAR-α/PGC-1α, which is dysregulated in AFLD [24, 25]. As shown in Fig. 4A–B, both Hif-2α-siRNA and PT2399 promoted the expression of PPAR-α and PGC-1α at the protein level, which allowed for direct comparison of the EtOH-treated group. Subsequently, in order to evaluate the effect of mitophagy on the PPAR-α/PGC-1α signaling pathway, pc-DNA3.1-BNIP3 was transfected into EtOH-treated AML-12 cells to overexpress BNIP3, resulting in higher levels of mitophagy-associated proteins in the pc-DNA3.1-BNIP3 treatment group compared to that in the control (Additional file 1: Figure S4A). Furthermore, pc-DNA3.1-BNIP3 treatment increased the colocalization of LC3 and MTR and the number of fluorescent puncta corresponding to BNIP3 and LC3 (Additional file 1: Figure S4C, D). In particular, the expression of PPAR-α/PGC-1α signaling pathway-related proteins and key enzymes for fatty acid oxidation, such as MCAD and CPT-1α, was higher in the pc-DNA3.1-BNIP3 treatment group (Fig. 4C, D), and was reduced in the Hif-2α-siRNA and BNIP3-shRNA co-treatment groups (Fig. 4E, Additional file 1: Figure S4B), compared to that in the control group. Based on these results, the PPAR-α/PGC-1α signaling pathway, which is involved in hepatic fatty acid β-oxidation and is essential for blocking the progression of AFLD, is negatively regulated by BNIP3-mediated mitophagy.

## Discussion

Alcohol consumption can lead to the occurrence of ALD, including AFLD, alcoholic fibrosis, and alcoholic cirrhosis [1, 2]. AFLD, which is characterized by lipid accumulation in hepatocytes, is the earliest stage of ALD. Currently, the most effective treatment for AFLD is alcohol abstinence, and more specific pharmacological treatments have not been fully explored or established [26]. New therapies are therefore urgently needed to prevent the progression of AFLD, especially for patients who cannot stop consuming alcohol.

Liver steatosis is a complex process, and fatty acid β-oxidation plays an indispensable role in it [27, 28]. Clinical research has shown that hepatic lipid synthesis is upregulated in most NAFLD patients, with many presenting with defective fatty acid β-oxidation [29]. In the present study, we observed a reduced expression of two key enzymes associated with hepatic fatty acid β-oxidation, CPT-1α and MCAD, both in vitro and in vivo, accompanied by increased lipid droplet numbers and triglyceride levels. The observed reduction in hepatic fatty acid β-oxidation is therefore related to ethanol-induced liver steatosis. Based on this, reversing fatty acid β-oxidation dysfunction could be important for the prevention and treatment of AFLD.

Multiple pathologic conditions can induce tissue hypoxia, which is an important mediator of lipid metabolism [30, 31]. Chronic and acute alcohol intake stimulates hepatic oxygen consumption, and subsequently causes hepatic hypoxia, leading to Hif pathway activation. Both human and murine studies have reported that the constitutive activation of Hif-2α during steatosis is associated with impaired fatty acid oxidation, increased lipid storage capacity, and serum triglyceride levels [20, 32–34]. Therefore, the Hif-2α pathway is increasingly becoming recognized as a vital mediator of lipid metabolism in the liver, although its potential molecular mechanisms in AFLD remain unclear. In the present study, we found that Hif-2α levels were higher in the liver tissue from EtOH-fed mice and EtOH-treated AML-12 cells. We also found that PT2399 [22, 23], a specific inhibitor of Hif-2α, could upregulate the levels of CPT-1α and MCAD, both in vitro and in vivo. In addition, Oil red o and H&E staining revealed a significant reduction in the level of liver injury and steatosis in EtOH-fed mice after PT2399 treatment. These observations confirmed the role of Hif-2α in AFLD progression and showed that attenuated fatty acid β-oxidation would occur as a consequence of EtOH-induced accumulation of Hif-2α protein.

PPAR-α is the main transcription factor for genes involved in oxidation, transport, and export of fatty acids. PGC-1α is an auxiliary activator of PPAR-α, and can jointly enhance fatty acid oxidation. During the development of renal fibrosis, blocking the PPAR-α/PGC-1α signaling pathway can efficiently deplete the key enzymes that are responsible for fatty acid β-oxidation, and thus aggravate the degree of kidney disease [35, 36]. In our present study, we demonstrated the increased expression of PPAR-α and PGC-1α in the absence of Hif-2α in EtOH-treated AML-12 cells, suggesting that there is a negative correlation between Hif-2α and the PPAR-α/PGC-1α signaling pathway in response to EtOH-induced hepatic lipid metabolism dysfunction.

Some studies have shown that ethanol blocks mitophagy by damaging lysosomes, but others have found that acute alcohol consumption can induce mitophagy [37–39]. In the present study, we used TEM to analyze liver tissue from EtOH-fed mice and observed increased numbers of lipid vacuoles but decreased autophagosomes compared to those in the control liver tissue. Upon EtOH feeding, the expression of mitophagy-associated genes BNIP3, Beclin1, and LC3II was reduced at the mRNA and protein levels, which was in agreement with our in vitro results. We also observed attenuated colocalization of LC3 and MTR in EtOH-treated AML-12 cells, accompanied by fewer fluorescent puncta corresponding to BNIP3 and LC3. These results suggest that ethanol can impair mitochondrial morphology and mitophagy capacity in the liver. Previous studies have established that the survival of autophagocytes in hepatic mitochondria is mainly regulated by BNIP3, and that the loss of BNIP3 causes liver injury and facilitates lipid accumulation [40, 41]. We therefore investigated the overexpression of BNIP3 in vitro to determine its role in hepatic mitophagy. As expected, transfection with pc-DNA3.1-BNIP3 resulted in the accumulation of mitophagy-associated proteins in EtOH-treated AML-12 cells, which is in agreement with previous findings. Somewhat surprisingly, we also found that the expression patterns of PPAR-α/PGC-1α signaling pathway-related proteins and two key enzymes concerning fatty acid β-oxidation were significantly reversed following transfection with pc-DNA3.1-BNIP3. We therefore concluded that mitophagy, which is under the control of BNIP3, exerts a significant influence on hepatic fatty acid β-oxidation, possibly via activating the PPAR-α/PGC-1α signaling pathway. Importantly, PT2399-mediated inhibition of Hif-2α resulted in fewer lipid vacuoles and more autophagosomes in vivo, and notably upregulated expression of BNIP3, Beclin1, and LC3II at the protein level both in vivo and in vitro. Hence, there may be crosstalk between Hif-2α and BNIP3-dependent mitophagy, and the role of Hif-2α may explain the observed BNIP3-dependent mitophagy even with EtOH stimulation.

**Fig. 5:**
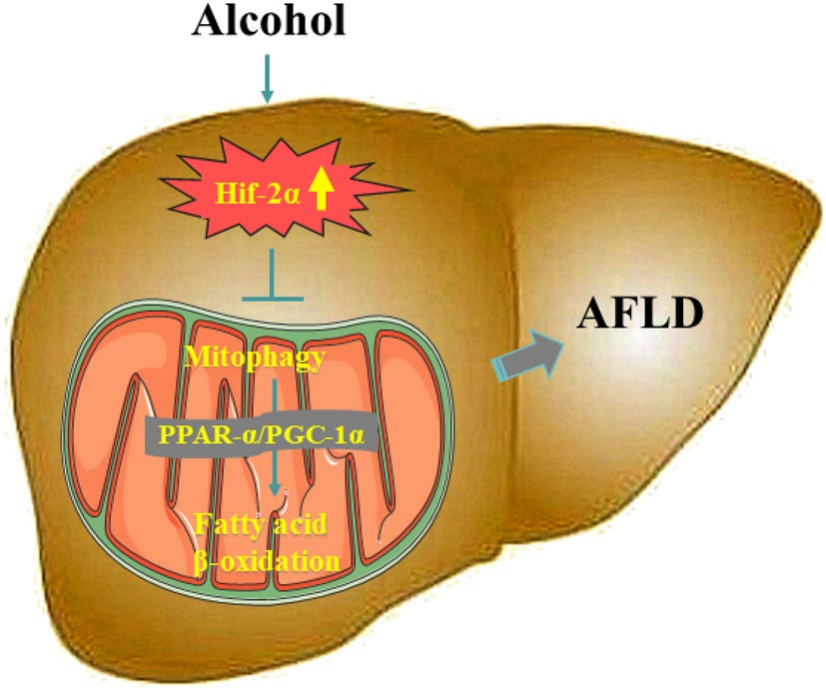
Model describing the pivotal role played by Hif-2α in AFLD development. EtOH-exposed fatty liver tissue has higher levels of Hif-2α, which subsequently attenuate the expression of many target genes, including those directing mitophagy and fatty acid β-oxidation, thereby accelerating AFLD development

In conclusion, the present study may contribute to a better understanding of the molecular basis of lipid accumulation in AFLD. First, abnormally high expression of Hif-2α is associated with EtOH stimulation, both in vivo and in vitro. By limiting Hif-2α expression, we enhanced fatty acid β-oxidation and mitophagy in EtOH-fed mice and EtOH-treated AML-12 cells. Taken together, we have presented evidence supporting the negative relationship between Hif-2α and the PPAR-α/PGC-1α signaling pathway and that it involves targeting BNIP3-dependent mitophagy, thus participating in hepatic lipid metabolism (Fig. 5). Our findings provide new pharmacological evidence that Hif-2α and its associated pathways should be carefully considered as therapeutic targets for AFLD.

## Conclusion

Our observations emphasize the importance of Hif-2α in promoting the progression of liver steatosis by functioning as a negative regulator of hepatic fatty acid β-oxidation and BNIP3-dependent mitophagy. We determined that Hif-2α regulates the PPAR-α /PGC-1α signaling pathway, which is related to hepatic fatty acid β-oxidation, in a BNIP3-dependent mitophagy-dependent manner. The present study provides a scientific basis for targeted therapy of AFLD via Hif-2α and its related pathways.

## Supplementary Information

**Additional file 1: Figure S1.** (A) Liver-to-body ratio, serum ALT, AST, T-CHO, and TG levels in liver tissue from EtOH-fed mice. (B, C) Representative images of H&E staining in liver tissue from EtOH-fed and PT2399-treated mice (scale bar = 100 μm or 20 μm) (n = 6). (D) Western blotting analysis of Hif-2α expression in the nucleus and cytoplasm in EtOH-treated AML-12 cells. Representative images of Hif-2α (green) and nuclei (blue) in EtOH-treated AML-12 cells (scale bar = 15 μm) (n = 3). Data are presented as the mean ± S.D. *P < 0.05, **P < 0.01. **Figure S2.** (A) Triglyceride (TG) levels in EtOH-treated AML-12 cells. (B) MTT assay, the level of TGs, and qRT-PCR analysis of VEGF expression in AML-12 cells at different concentrations of PT2399 (n = 3). (C) Liver-to-body ratio, serum T-CHO, ALT, and AST levels in the liver tissue of PT2399-treated mice (n = 6). (D, E) Western blotting analysis of BNIP3, Beclin1, and LC3II/LC3I expression in EtOH-treated AML-12 cells, and Hif-2α-siRNA and BNIP3-shRNA co-treated AML-12 cells (n = 3). Data are presented as the mean ± S.D. *P < 0.05, **P < 0.01. **Figure S3.** (A) Representative images of mitochondria (red), LC3 (green), and nuclei (blue) in EtOH-treated AML-12 cells. (B, C) Representative images of BNIP3 (red), LC3 (green), and nuclei (blue) in EtOH-treated and PT2399-treated AML-12 cells (scale bar = 15 μm) (n = 3). (D) Representative TEM images of lipid droplets (LDs), mitochondria, and autophagosomes in the liver tissue of EtOH-fed mice. Black arrows denote autophagosomes, yellow arrows denote LDs, and red arrows denote mitochondria (n = 6). Data are presented as the mean ± S.D. *P < 0.05, **P < 0.01. **Figure S4.** (A) Western blotting analysis of BNIP3, Beclin1, and LC3II/LC3I expression in pc-DNA3.1-BNIP3-treated AML-12 cells. (B) Western blotting analysis of CPT-1α and MCAD expression in Hif-2a-siRNA and BNIP3-shRNA co-treated AML-12 cells (n = 3). Data are presented as the mean ± S.D. *P < 0.05, **P < 0.01. (C) Representative images of mitochondria (red), LC3 (green), and nuclei (blue) in pc-DNA3.1-BNIP3-treated AML-12 cells. (D) Representative images of BNIP3 (red), LC3 (green), and nuclei (blue) in pc-DNA3.1-BNIP3-treated AML-12 cells (scale bar = 15 μm).

## Data Availability

All supporting data are included in the main article and its supplementary files are available from the corresponding author upon request.

## Abbreviations

AFLD: Alcoholic fatty liver disease
ALD: Alcoholic liver disease
ASH: Alcoholic steatohepatitis
HCC: Hepatocellular carcinoma
CPT-1: Carnitine-palmitoyl transferase-1
Hif: Hypoxia inducible factor
Hif-2α: Hypoxia inducible factor-2α
H&E: Hematoxylin and eosin
IHC: Immunohistochemistry
ALT: Alanine aminotransferase
AST: Aspartate aminotransferase
Pparα: Peroxisome proliferators activated receptor α
LC3B: Microtubule-associated light chain 3B
Beclin1: Programmed cell death-1
BNIP3: BCL2/adenovirus E1B 19 kDa Interacting protein 3
TG: Triglyceride
T-CHO: Total cholesterol
siRNA: Small interfering RNA
shRNA: Short hairpin RNA
