# Supplementary material for: Hif-2α regulates lipid metabolism in alcoholic fatty liver disease through mitophagy
Source: Cell Biosci. 2022 Dec 7;12:198. doi: 10.1186/s13578-022-00889-1 (PMC9730692; doi:10.1186/s13578-022-00889-1)
Supplement: Supplementary file 1 — Additional file 1: Figure S1. (A) Liver-to-body ratio, serum ALT, AST, T-CHO, and TG levels in liver tissue from EtOH-fed mice. (B, C) Representative images of H&E staining in liver tissue from EtOH-fed and PT2399-treated mice (scale bar = 100 μm or 20 μm) (n = 6). (D) Western blotting analysis of Hif-2α expression in the nucleus and cytoplasm in EtOH-treated AML-12 cells. Representative images of Hif-2α (green) and nuclei (blue) in EtOH-treated AML-12 cells (scale bar = 15 μm) (n = 3). Data are presented as the mean ± S.D. *P < 0.05, **P < 0.01. Figure S2. (A) Triglyceride (TG) levels in EtOH-treated AML-12 cells. (B) MTT assay, the level of TGs, and qRT-PCR analysis of VEGF expression in AML-12 cells at different concentrations of PT2399 (n = 3). (C) Liver-to-body ratio, serum T-CHO, ALT, and AST levels in the liver tissue of PT2399-treated mice (n = 6). (D, E) Western blotting analysis of BNIP3, Beclin1, and LC3II/LC3I expression in EtOH-treated AML-12 cells, and Hif-2α-siRNA and BNIP3-shRNA co-treated AML-12 cells (n = 3). Data are presented as the mean ± S.D. *P < 0.05, **P < 0.01. Figure S3. (A) Representative images of mitochondria (red), LC3 (green), and nuclei (blue) in EtOH-treated AML-12 cells. (B, C) Representative images of BNIP3 (red), LC3 (green), and nuclei (blue) in EtOH-treated and PT2399-treated AML-12 cells (scale bar = 15 μm) (n = 3). (D) Representative TEM images of lipid droplets (LDs), mitochondria, and autophagosomes in the liver tissue of EtOH-fed mice. Black arrows denote autophagosomes, yellow arrows denote LDs, and red arrows denote mitochondria (n = 6). Data are presented as the mean ± S.D. *P < 0.05, **P < 0.01. Figure S4. (A) Western blotting analysis of BNIP3, Beclin1, and LC3II/LC3I expression in pc-DNA3.1-BNIP3-treated AML-12 cells. (B) Western blotting analysis of CPT-1α and MCAD expression in Hif-2a-siRNA and BNIP3-shRNA co-treated AML-12 cells (n = 3). Data are presented as the mean ± S.D. *P < 0.05, **P < 0.01. (C) Representati [file 13578_2022_889_MOESM1_ESM.pptx]

## Slide 1
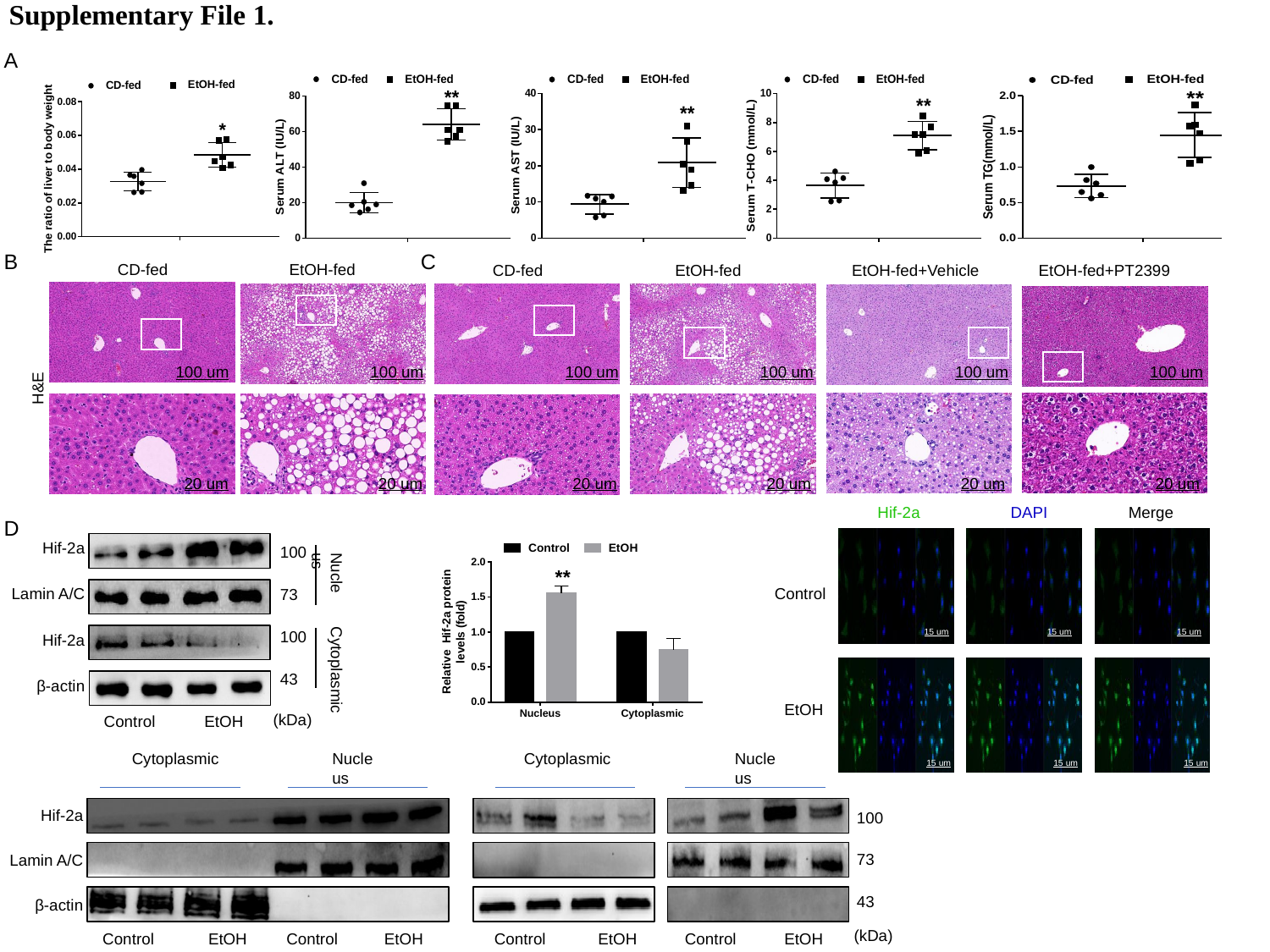

Supplementary File 1.
A
B
C
CD-fed
EtOH-fed
100 um
100 um
20 um
20 um
H&E
CD-fed
EtOH-fed
EtOH-fed+Vehicle
EtOH-fed+PT2399
100 um
100 um
100 um
100 um
20 um
20 um
20 um
20 um
Hif-2a
DAPI
Merge
Control
EtOH
15 um
15 um
15 um
15 um
15 um
15 um
D
Hif-2a
Lamin A/C
Hif-2a
Control
100
73
43
(kDa)
100
β-actin
 EtOH
Nucleus
Cytoplasmic
Cytoplasmic
Cytoplasmic
Nucleus
Nucleus
Hif-2a
Lamin A/C
β-actin
 EtOH
Control
Control
Control
Control
 EtOH
 EtOH
 EtOH
100
73
43
(kDa)
15 um
15 um
15 um

## Slide 2
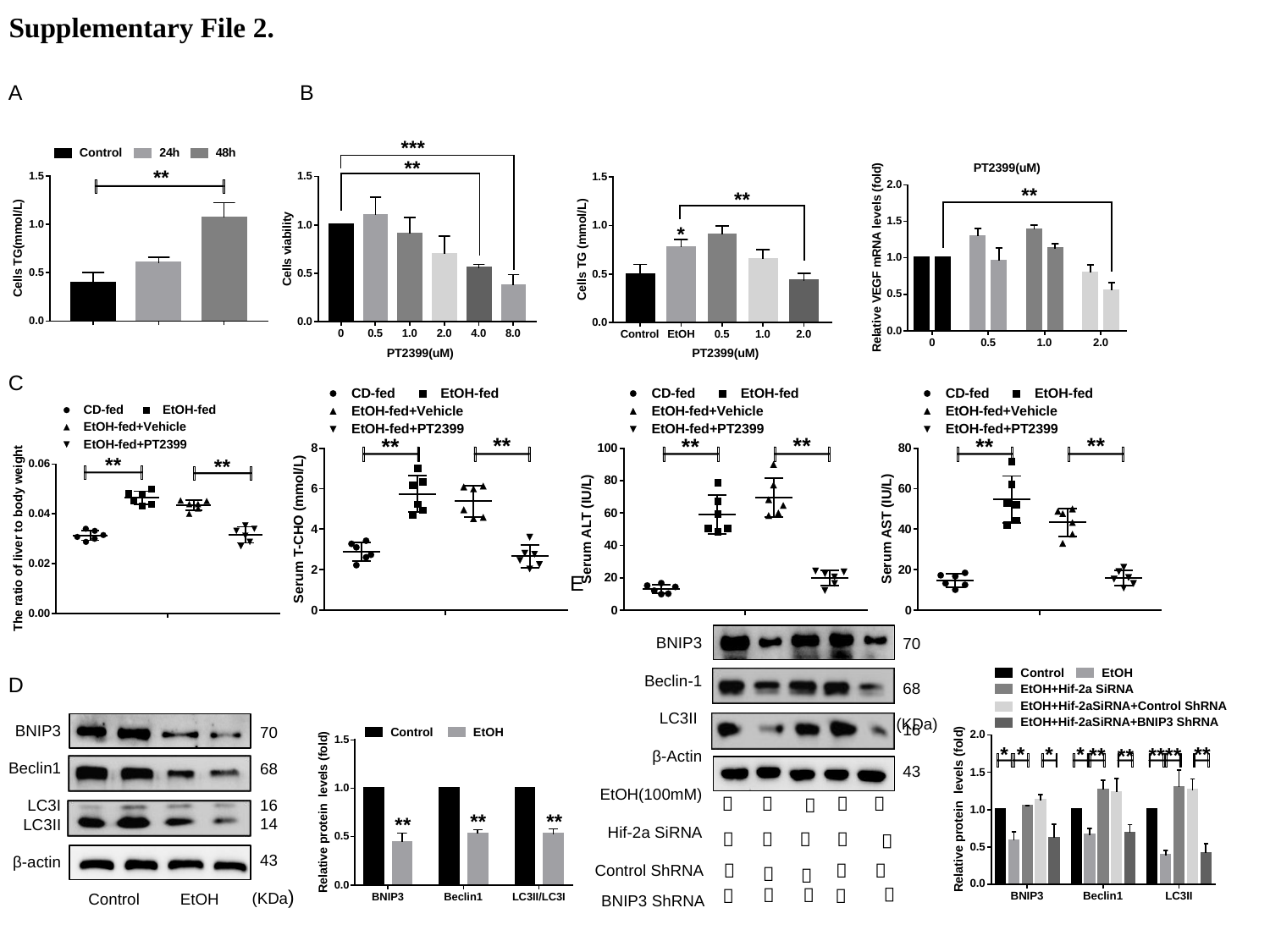

Supplementary File 2.
A
B
C
E
BNIP3
Beclin-1
β-Actin
＋
－
＋
－
－
－
＋
－
－
－
－
－
－
＋
＋
＋
＋
＋
＋
－
70
68
16
43
LC3II
 EtOH(100mM)
Hif-2a SiRNA
BNIP3 ShRNA
Control ShRNA
D
(KDa)
BNIP3
Beclin1
β-actin
LC3I
LC3II
Control
68
16
14
43
(KDa)
70
 EtOH

## Slide 3
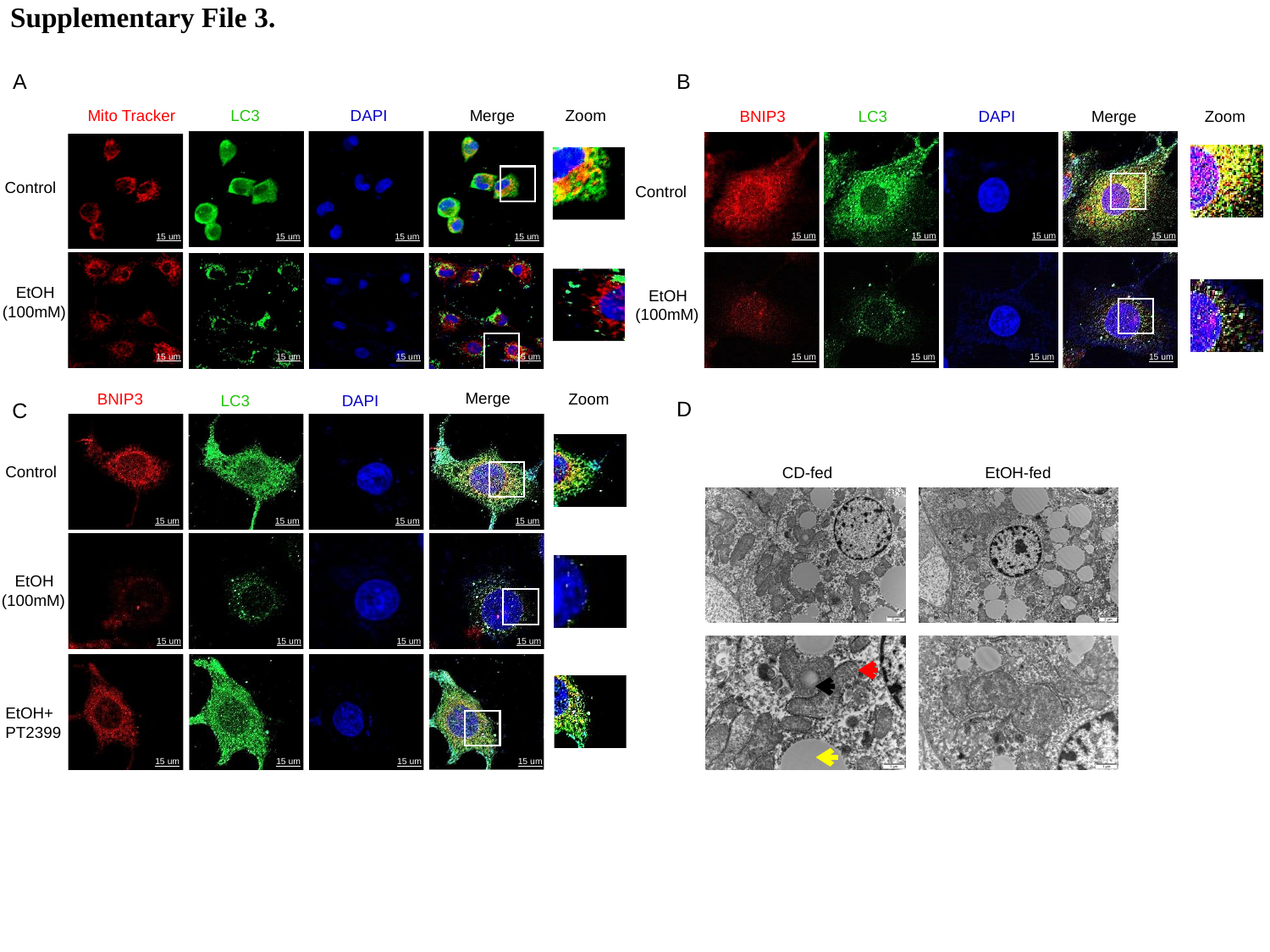

Supplementary File 3.
A
B
LC3
Merge
Control
DAPI
Zoom
 EtOH
(100mM)
Mito Tracker
BNIP3
LC3
DAPI
Merge
Control
 EtOH
(100mM)
Zoom
15 um
15 um
15 um
15 um
15 um
15 um
15 um
15 um
15 um
15 um
15 um
15 um
15 um
15 um
15 um
15 um
Merge
BNIP3
LC3
DAPI
Control
 EtOH
(100mM)
EtOH+
PT2399
Zoom
D
C
15 um
CD-fed
EtOH-fed
15 um
15 um
15 um
15 um
15 um
15 um
15 um
15 um
15 um
15 um
15 um
15 um
15 um
15 um
15 um
15 um

## Slide 4
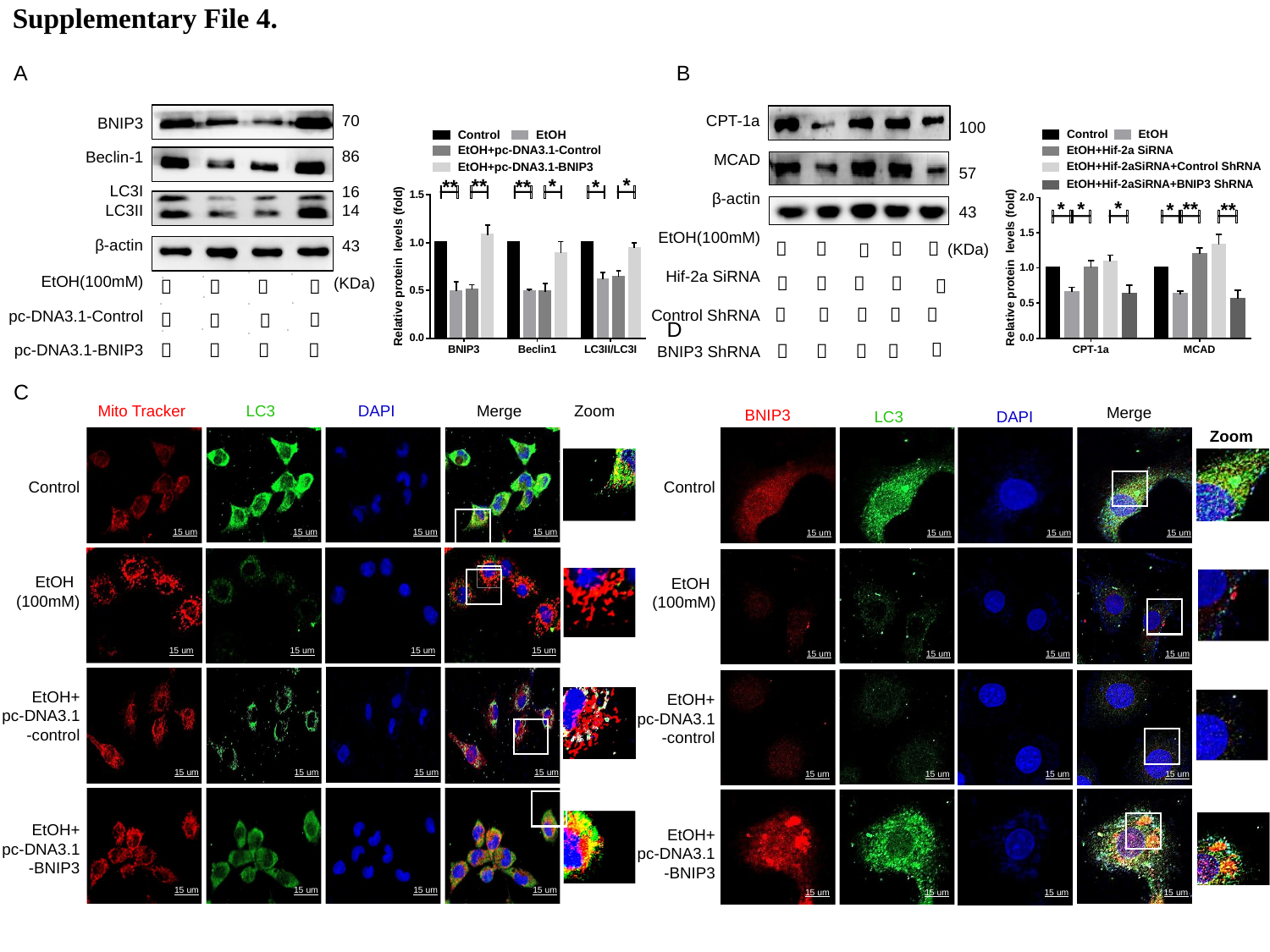

Supplementary File 4.
A
B
70
16
14
43
(KDa)
86
BNIP3
Beclin-1
LC3I
LC3II
β-actin
－
－
＋
pc-DNA3.1-Control
－
＋
－
pc-DNA3.1-BNIP3
－
－
＋
＋
＋
－
 EtOH(100mM)
－
－
－
－
＋
－
－
－
＋
＋
＋
＋
CPT-1a
β-actin
＋
－
＋
－
－
－
＋
－
－
－
－
－
－
＋
＋
＋
＋
＋
＋
－
57
43
MCAD
 EtOH(100mM)
Hif-2a SiRNA
BNIP3 ShRNA
Control ShRNA
(KDa)
100
D
C
LC3
DAPI
Merge
Zoom
Control
 EtOH
(100mM)
EtOH+
pc-DNA3.1
-control
EtOH+
pc-DNA3.1
-BNIP3
Mito Tracker
Merge
BNIP3
LC3
DAPI
Control
 EtOH
(100mM)
EtOH+
pc-DNA3.1
-control
EtOH+
pc-DNA3.1
-BNIP3
15 um
15 um
15 um
15 um
15 um
15 um
15 um
15 um
15 um
15 um
15 um
15 um
15 um
15 um
15 um
15 um
15 um
15 um
15 um
15 um
15 um
15 um
15 um
15 um
15 um
15 um
15 um
15 um
15 um
15 um
15 um
15 um
Zoom
